# Predicting Critical Micelle Concentrations from Short Time Scale Simulations

**DOI:** 10.1021/acs.jpcb.5c07475

**Published:** 2025-12-17

**Authors:** Felix Rummel, Joshua F. Robinson, Patrick B. Warren, David J. Bray, Richard L. Anderson

**Affiliations:** † The Hartree Centre, STFC Daresbury Laboratory, Warrington WA4 4AD, U.K.; ‡ H. H. Wills Physics Laboratory, University of Bristol, Bristol BS8 1TL, U.K.

## Abstract

The determination of surfactant critical micelle concentrations (CMCs) using molecular simulations is by now a well-established procedure. The approach typically utilizes the equilibrium free surfactant concentration (i.e., those surfactants not in micelles). However, equilibration can be a slow process, particularly for surfactants with low CMC values, requiring large amounts of compute resource even when coarse grained approaches are adopted. We here explore the possibility of using incompletely equilibrated short time scale runs to predict surfactant CMCs using dissipative particle dynamics (DPD) simulations. We ground the methodology in the Aniansson–Wall stepwise association model of micelle kinetics, and apply it to a selection of uncharged (nonionic and zwitterionic) and charged (ionic) surfactants. We find the approach to be useful for predicting the CMC of both charged and uncharged surfactants.

## Graphical abstract

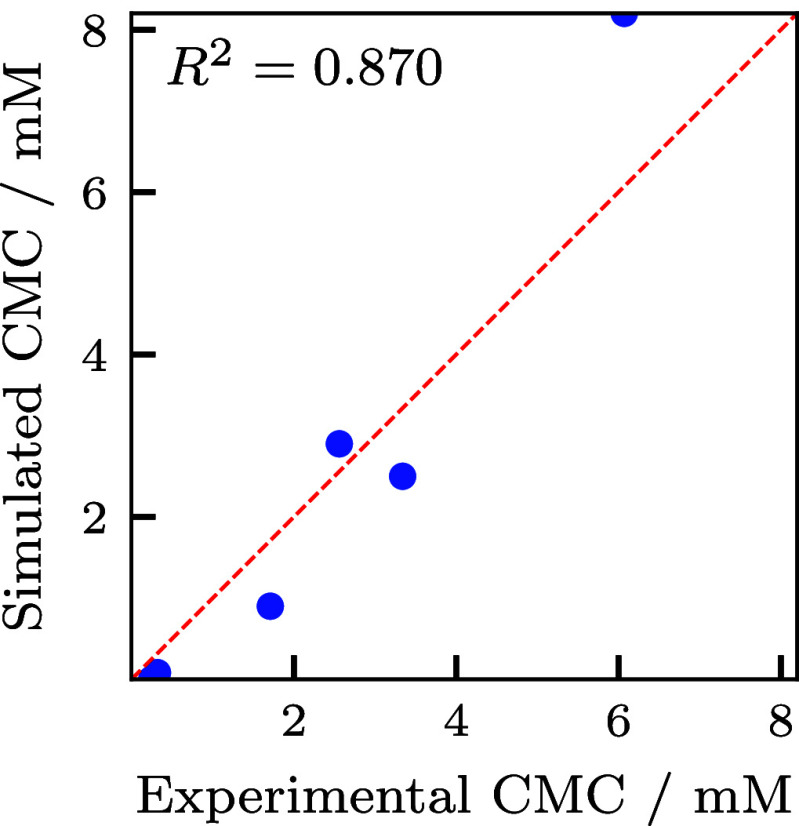

## 1 Introduction

Surfactants are ubiquitous across multiple industries, from manufacturing processes to household and personal care products. When placed in water, surfactants spontaneously organize into supramolecular structures called micelles. The concentration at which micelles first emerge is known as the critical micelle concentration (CMC). In product formulation, the CMC is an important design target as it is directly related to many physicochemical properties such as a minimum in interfacial tension,^1^ and a maximum in detergency.^2^ Given its importance, there has been a concerted effort to develop thermodynamic approaches, e.g. COSMO-type models,^3,4^ and simulation approaches, e.g. dissipative particle dynamics (DPD) models,^5–9^ other molecular dynamics, simulations^10,11^ or machine learning based approaches,^12^ capable of accurately and efficiently predicting the CMC. Where accurate models exist, computational methods may expedite the discovery of novel surfactants alongside laboratory-based programmes. Such models have become increasingly important against the backdrop of a shift toward sustainable sourcing in the multibillion dollar surfactant market necessitating a reevaluation of surfactant choice.

In simulation approaches, the calculation of the CMC can be based on measuring the so-called “free” or unassociated surfactant concentration, which can be readily done by applying a clustering algorithm to distinguish “free” surfactants in monomers and submicellar aggregates from those in micelles.^5,8^ We have previously used DPD to obtain CMC values this way for a variety of surfactants,^8,13^ however this approach is predicated on the system being well-equilibrated, in the sense that the free surfactant concentration has reached a steady-state value. Equilibration can be a slow process, particularly for surfactants with small CMCs, rendering the simulation approach prohibitively expensive in terms of compute resource. DPD is particular advantageous over conventional simulations because its coarse-grained approach makes longer length and time scales accessible within the same compute time.^14^ Other approaches based on viscosity or interfacial tensions also exist.^10,11^ These, however, encounter similar challenges with slow equilibration rendering them computationally expensive. In the case of interfacial tension calculations this can be especially problematic due to electrostatic repulsion between surfactants at the interface and in solution.

We here examine the possibility of using replicate short time scale simulation runs to predict the CMC of surfactants using DPD simulations. Our approach is based on the notion that the final steady-state free surfactant concentration can be reliably and accurately predicted, once enough data has been accumulated from such runs. The analysis is underpinned by the Aniansson–Wall stepwise association model of surfactant self-assembly,^15^ which can be used not only for describing micellar kinetics in the equilibrium state, but also to describe the approach to steady state, motivating the fitting procedures for this approach.

The article is arranged as follows. In the next section (Section 2) we discuss the thermodynamics of micellization. This forms the framework for the following section (Section 3) where we discuss the theory of micellization kinetics. In Section 4 we describe the coarse-grained DPD model employed to simulate the surfactants of this study. We also describe the protocol developed to calculate the CMC of these surfactants from (relatively) short time scale simulations and describe the specific details of the simulations performed. Our results are presented in Section 5 where we explore different protocols of using short time scale simulations to maximize accuracy while minimizing computational cost. Finally, in Section 6 we discuss the results and summarize the potential for future developments.

## 2 Theory of Micelle Formation

We start with an illustrative model due to Maibaum, Dinner and Chandler (MDC) for the change in the (excess) free energy due to aggregating *n* surfactant molecules out of a monomer bath as^16^

1 $$G_{n}^{ex}-nG_{1}^{ex}≃-g\left(n-1\right)+\gamma {\left(n-1\right)}^{2/3}+h{\left(n-1\right)}^{5/3}$$

Here *G*_1_^ex^ is the excess free energy of a single monomer, and we work in units of the thermal energy *k*_B_ *T*. The coefficients *g* and γ are the respective bulk and surface tension terms of standard classical nucleation theory,^17^ with *g* > 0 providing the driving force for aggregation. These are augmented by the stoichiometric term with coefficient *h* that accounts for the colocalization of head and tail groups.^18^ This superextensive penalty suppresses unbounded aggregate growth. The concentration of aggregates relative to the monomer follows as $c_{n}\Lambda^{3}=e^{-W_{n}}$ where *W*_*n*_ ≡ *G*_*n*_^ex^ – *nG*_1_ plays the role of a potential of mean force and Λ is a natural length unit (e.g., monomer size). The ideal gas term in *G*_1_ = *G*_1_^id^ + *G*_1_^ex^ effectively transforms *g* → *g* + ln­(*c*_1_Λ^3^), so increasing the concentration drives aggregation as

2 $$c_{n}=K_{n}c_{1}^{n}$$

where *c*_1_ and *c*_*n*_ are the number densities of monomers and *n*-mers respectively, and *K*_*n*_ ∼ exp­[−(*G*_*n*_^ex^ – *nG*_1_^ex^)]. This quasi-chemical equilibrium interpretation aligns with the kinetic models discussed in the next section.

The CMC can then be interpreted as the monomer concentration above which the free energy *W*_*n*_ develops a saddle at *n* = n* as shown in Figure 1a. Above the CMC, the aggregate distribution becomes increasingly bimodal as shown in Figure 1b. While this theory is qualitatively helpful, directly measuring the free energy *G*_*n*_^ex^ in simulation can be very expensive and so more indirect methods are required to efficiently determine the CMC as discussed in section Section I.

**Figure 1:**
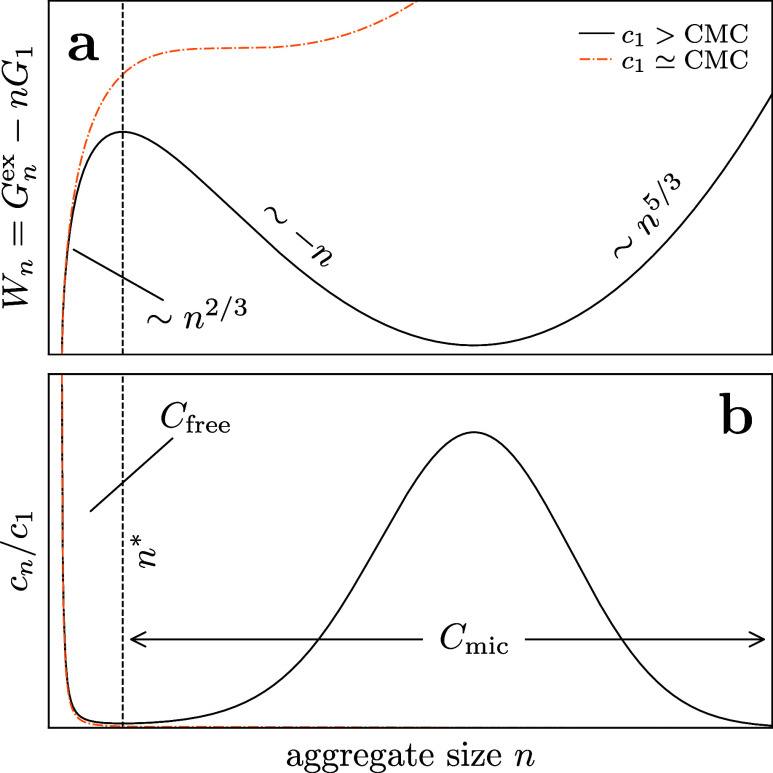
(color online) Sketch of distribution of aggregates of *n* surfactant molecules. Above the critical micelle concentration (CMC) stable micelles form with *n* > n*. (a) Effective energy landscape following model eq 1 and (b) the resulting concentration of the aggregates.

Assuming a particular saddle value n*, it is possible to determine the concentration of submicellar “free” surfactant $C_{free}=\sum_{n<n^{*}}c_{n}$ , and the concentration of micellar surfactant $C_{mic}=\sum_{n>n^{*}}c_{n}$ , as illustrated in Figure 1b. The total surfactant concentration (a constraint) then follows *C*_tot_ = *C*_free_ + *C*_mic_. If the two regions overlap n* can be sleuthed from the minimum in *c*_*n*_.^19^ For other systems where *c*_*n*_ ≃ 0 around n*, the partitioning into *C*_free_ and *C*_mic_ is insensitive to the particular choice of n* and a suitable value can be chosen pragmatically. With knowledge of the values for *C*_tot_ and *C*_free_ the CMC can be computed using the methodologies described below.

In a simple approach, one may set up a series of simulations to determine *C*_free_ at several increasing values of *C*_tot_. One then has for nonionic surfactants

3 $$C_{free}≃CMC$$

at concentrations *C*_tot_ > CMC. The CMC can then be assigned to the plateau/average value of *C*_free_, as *C*_tot_ is increased above the CMC. This approach assumes that as more surfactant molecules are added to a simulation box, these only go toward building more micelles so that *C*_free_ remains constant reflecting the approximate constant surfactant chemical potential.^8,13,20^ This is an oversimplification since the value of *C*_free_ does not (necessarily) remain constant as *C*_tot_ is increased above the surfactant’s CMC.^21–26^ In the case of nonionic surfactants, one must consider the reduction in accessible volume for the free surfactant. This effect can be accounted for by factoring in the volume (packing fraction) occupied by surfactant in the simulation box in determination of CMC. Alternatively, one can sample at multiple concentrations only just above the CMC where any crowding effects are minimized. This is described in detail in the appendix of Anderson et al.^8^

For ionic surfactants, there is a significant and systematic depression in *C*_free_ as one increases the total surfactant concentration above the CMC. This arises because adding more surfactant also adds more counterions, which depresses the free surfactant concentration as a kind of common ion effect. The phenomenology can be captured in so-called “charge phase models” of micelle formation from which one can show that^27–29^

4 $$\begin{array}{l} \log C_{free}+\beta \;\log\left(\frac{\beta C_{free}+\left(1-\beta \right)C_{tot}}{\left(1-\phi \right)}\right)\\ ≃\left(1+\beta \right)\log CMC \end{array}$$

The factor 1/(1 – ϕ) adjusts for the crowding effect on unassociated counterions where ϕ = *V*_m_ *C*_tot_ is the surfactant packing fraction, and *V*_m_ is the surfactant molar volume, which we calculate for each surfactant assuming a density of 1 g/cm^3^. The variable β in eq 4 describes the degree of counterion binding to micelles, which we note reduces to eq 3 for β = 0. Equation 4 has been applied by several authors to determine CMC from simulation.^6,8,23,30,31^

While undoubtedly successful, a drawback of this method is the relatively long simulation times required to achieve a reliable CMC from simulation. Simulations can take many millions of time steps and the associated wall-clock time can take several days (depending upon the computational resources at the scientists disposal). They must be run long enough to ensure that *C*_free_, and therefore concentration of surfactants in micelles are well equilibrated (i.e., *t* > *t*_e_: d*C*_free_/d*t* ≃ 0 where *C*_free_ is the nonaggregated surfactant concentration). The time taken to achieve this, *t*_e_, varies with a number of variables such as box size, surfactant concentration and surfactant chemistry. In our works we have reported upon systems that take several million simulation time steps to achieve satisfactory steady-state conditions by this criterion.^13^

## 3 Theory of Micellization Kinetics

### 3.1 Aggregation Model

A coarse-grained view of aggregation kinetics can be obtained by imagining the general quasi-chemical process of stepwise association/dissociation

5 $$A_{1}+A_{n}\overset{k_{n}^{+}}{\underset{k_{n}^{-}}{⇌}}A_{n+1}$$

in terms of aggregates *A*_*n*_ (with *A*_1_ being the monomer) and where *k*_*n*_^±^ are the rate constants for the forward/backward aggregation processes. This is the Becker–Döring kinetic nucleation model,^32^ more commonly known in this context as the Aniansson–Wall stepwise association model.^15^

The net chemical flux from *n* → *n* + 1 is

$$j_{n}=k_{1,n}^{+}c_{1}c_{n}-k_{1,n}^{-}c_{n+1},\forall n\geq 1$$

where *c*_*n*_ = [*A*_*n*_]. This quantity vanishes in equilibrium *c*_*n*_ = *c*_*n*_^eq^ giving the condition

$$\frac{k_{n}^{+}}{k_{n}^{-}}=\frac{c_{n+1}^{eq}}{c_{n}^{eq}c_{1}^{eq}}$$

For the equilibrium concentrations *c*_*n*_^eq^ we use the MDC model for micelles eq 1. Strictly speaking this model only applies for nonionic surfactants, but qualitatively it suffices as the basic bimodal shape of *c*_*n*_^eq^ remains the same for ionic surfactants. For simplicity we assume the forward kinetics are diffusion limited,^33^ i.e.

6 $$\begin{array}{l} k_{n}^{+}\propto \left(R_{1}+R_{n}\right)\left(D_{1}+D_{n}\right)\\ \propto \left(R_{1}+R_{n}\right)\left(\frac{1}{R_{1}}+\frac{1}{R_{n}}\right)\\ =\left(1+n^{1/3}\right)\left(1+n^{-1/3}\right) \end{array}$$

where *D*_*n*_ ∝ 1/*R*_*n*_ is the diffusion coefficient of an aggregate of size *n* assuming Stokes–Einstein and approximately spherical aggregates, and *R*_*n*_ ∝ *n* ^1/3^ is the radius. Note that we set the prefactor to unity in the final line here effectively setting the dimer formation rate *k*_1_^+^ = 1/4. This (arbitrarily) fixes the units of time.

### 3.2 Long-Time Kinetics

The chemical rate equations have the form

$$\partial_{t}c=S\cdot V\left(c\right)$$

where **c** = ∑_*n*_ *c*_*n*_ **e**_*n*_ is a vector of aggregate number densities or concentrations, **S** is the stoichiometry matrix giving the participation of an aggregate of a given size in a given reaction, and **V** are the reaction velocities, which incorporate mass action kinetics for the quasi-chemical pathways of aggregation in eq 5. The results of directly simulating this system with M=1 (i.e., the standard Aniansson–Wall model of monomer exchange) starting from pure monomer solution are shown in Figure 2. The system rapidly forms micelles purple and orange curves in Figure 2a but with mean aggregation numbers ⟨*n*⟩ = ∑_*n*_ *nc*_*n*_/*C*_tot_ significantly lower than their equilibrium value. The final kinetic process involves a gradual increase of ⟨*n*⟩ over a much longer time scale than micelle formation. This slow time scale is conventionally called τ_2_, whereas the shorter time scales are generically represented by τ_1_.

**Figure 2:**
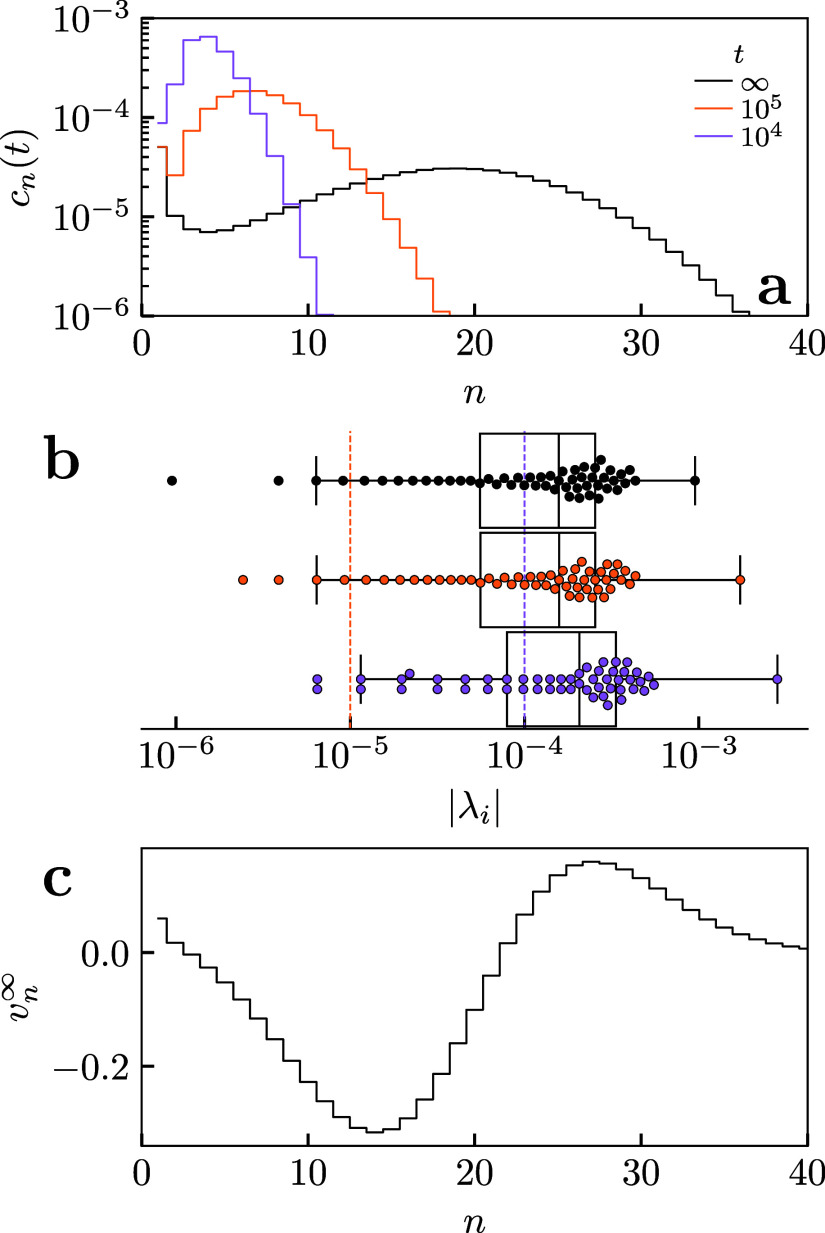
Theoretical prediction of micelle kinetics via Aniansson–Wall model. (a) Evolution in concentration of micelle aggregates of size *n* with time *t*. (b) Distribution of decay constants (inverse time) at sampled states in panel (a). (c) Longest time decay eigenvector represents final rearrangement during τ_2_ relaxation. In these runs we tuned the parameters entering the underlying thermodynamic model eq 1 so the peak of *c*_*n*_ occurs at *n* = 20, *c*_1_ = ϕ = 0.01 and assigning the parameter *h* consistent with a surfactant 5:1 length to girth ratio according to the theories of refs 16,18 .

The rates of distinct relaxation pathways can be obtained from the (inverse) right eigenvalues {λ_*i*_} of the instantaneous Jacobian of **S**·**V** with respect to the aggregate concentrations; this is a square matrix which we denote by **M**(*t*). We can track the evolution of the kinetic time scales and the emergence of the slow τ_2_ time scale by evaluating this matrix and solving for the associated eigenvalues along the trajectory of evolving aggregate concentrations. We show an example of the eigenvalues in Figure 2b. We see the emergence of a single slow pathway which is approximately an order of magnitude slower than the others, which we can interpret as the τ_2_ relaxation. However, multiple pathways are associated with the faster relaxation processes making τ_1_ less well-defined.

The right eigenvector of **M** associated with the slow τ_2_ pathway in equilibrium **v** ^∞^ is shown in Figure 2c. This eigenvector shows a decrease (increase) in aggregates with smaller *n* smaller (larger) than the equilibrium ⟨*n*⟩, confirming that this pathway corresponds to growth in the size of micelle aggregates i.e. increasing ⟨*n*⟩ toward its equilibrium value. This is consistent with the well-known^15^ emergence of the slow τ_2_ pathway corresponding to the quasi-conservation of micelles, since gaining or losing a micelle in a stepwise association process can only happen by condensation or evaporation from monomers, which involves a barrier-crossing event through the region of rare micelles, or the rare (but here neglected) fission or fusion of micelles.^34^ Of note is the fact that the amount of free monomer changes very little during the final relaxation *v*_1_^∞^ ≈ 0.

Approaching the steady-state concentration **c** ^eq^ the long-time kinetics is linearized as

$$\partial_{t}c=M^{eq}\left(c-c^{eq}\right)$$

where **M** ^eq^ ≡ ∇**R**|_**c** ^eq^_ is now a constant matrix Jacobian evaluated at equilibrium. The solution of this is exponential decay in the eigenmodes of **M** ^eq^

7 $$c=c^{eq}+\sum_{k>1}e^{-\lambda_{k}t}v_{k}$$

Here **v**_*k*_ are the right eigenvectors of **M** ^eq^ with eigenvalues {λ_*k*_} giving the rates. We exclude the zero right eigenmode with λ_1_ = 0 because this eigenvector represents a change in **c** from an increase in the surfactant concentration;^35^ here we assume constant concentration so this marginal mode must be ignored.

For the purposes of the short time simulations, our main point (from the linear stability analysis above) is that we generically expect the kinetics to follow an exponential decay, as in eq 7. To model equilibrium behavior we do not necessarily need to run simulations over times τ ≫ τ_2_; rather, we may be able to get away with running over a fraction of τ_2_ and infer equilibrium behavior by fitting the decay to an exponential. This is justified so long as the fitting is performed on a time scale τ ≫ τ_1_. To estimate the CMC using eq 4 we only need to know the free monomer concentration *C*_free_. As we saw in Figure 2c, the amount of free monomer *C*_free_ changes little during the τ_2_ relaxation further suggesting that CMC should be insensitive to partial τ_2_ relaxation. Together this suggests a time window τ_1_ ≪ τ ≪ τ_2_ should be sufficient to estimate τ via exponential fitting.

## 4 Methods

In this section we describe the computational approach adopted outlining the DPD models used to simulate the surfactants and the analysis performed. Details on the DPD methodology adopted and the model parameters utilized are given in the Supporting Information, which cites the following additional literature.^36–43^

### 4.1 The DPD Model

The DPD model adopted for this work follows our previously published works.^7,8,13,31,44,45^ Here, DPD beads represent chemical groups of 1 to 6 “heavy atoms” (e.g., carbon, oxygen, nitrogen, sulfur), except for water, which is treated supermolecularly with a water bead consisting of two water molecules (2H_2_O). This water bead mapping is used to infer a length scale mapping, which can then be used to convert simulation number densities and so on into physical concentration units.

In this work DPD bead volumes are informed by partial molar volumes, Durchschlag and Zipper^46^ and the interactions between DPD beads upon experimental log *P* values and liquid phase densities. As this method is now well established we do not give more extensive details here, rather we point to the cited prior literature and the Supporting Information which also contains details of the interaction parameters for the DPD simulations.

Following this model, the surfactants considered here are all linear chemically specific DPD bead chains, with structures as follows. Nonionic C_*n*_E_*m*_ alkyl ethoxylate surfactants comprise an alkyl chain of length *n* connected to an ethoxylate chain of length *m*, and are represented (for *n* even) by a block of CH_2_CH_2_ (alkyl) beads with a terminal CH_3_ (methyl) bead and a block of CH_2_OCH_2_ (ether) beads with a terminal CH_2_OH (alcohol) bead. The resulting coarse-grained C_*n*_E_*m*_ surfactant model is therefore

$$\left[{CH}_{3}\right]-{\left[{CH}_{2}{CH}_{2}\right]}_{n/2-1}-{\left[{CH}_{2}{OCH}_{2}\right]}_{m}-{CH}_{2}OH$$

where the square brackets denote the different bead types. For *n* odd (not considered here) the terminal CH_3_ bead could be replaced by a CH_3_CH_2_, as in our model of CAPB below. For SDS and SLE_*m*_S surfactants, we adopt a similar beading strategy. To model the sulfate moiety we adopt a bead comprised of 6 heavy atoms, i.e. CH_2_OSO_3_^–^, which carries a negative charge of −1. The counterion is modeled as Na^+^·2H_2_O (i.e., a partially hydrated sodium ion). The resulting bead structure for these surfactants is then (with *m* = 0 for SDS and *m* = 3 for SLE_3_S which we study here)

$$\left[{CH}_{3}\right]-{\left[{CH}_{2}{CH}_{2}\right]}_{5}-{\left[{CH}_{2}{OCH}_{2}\right]}_{m}-\left[{CH}_{2}{{OSO}_{3}}^{-}\right]\cdot \cdot \cdot \left[{Na}^{+}\cdot 2H_{2}O\right].$$

Similarly, CAPB follows

$$\left[{CH}_{3}{CH}_{2}\right]-{\left[{CH}_{2}{CH}_{2}\right]}_{4}-\left[{CH}_{2}C\left(=O\right){NHCH}_{2}\right]-\left[{CH}_{2}\right]-\left[{CH}_{2}N+{\left({CH}_{3}\right)}_{2}\right]-\left[{COO}^{-}\right]$$

We choose to only model the deprotonated (neutral) form of CAPB as this is the predominant form of this zwitterionic surfactant at its natural pH. In this case no counterion is needed.

### 4.2 CMC Prediction Algorithm

The initial stage of predicting the CMC from our simulations is the same as performed in previous works.^8,13^ Simulations are performed with the concentration of surfactant in the box at a level greater than the known CMC. During the simulation, surfactant molecules aggregate to form clusters which can be classified as micelles or submicellar. We use a cutoff of n* = 6 as the maximum size of a submicellar aggregate in this and our previous works. In principle, one could use different values for the cutoff for each system sampled once the distribution depicted in Figure 1 is determined. In practice, distributions resulting from DPD simulations are not so well formed and pragmatic choices for submicellar cutoff values are required. Once the distinction between submicellar and micellar aggregates is made the total number of micelles and their corresponding aggregation number (*n*) can be extracted, along with the amount of submicellar aggregates (which includes free monomers) from simulation trajectories for each time step. From these values we can determine the ratio of *C*_free_ to *C*_mic_.

To calculate the CMC we took two approaches: the established method where we ran and evaluated simulations at various concentrations, just above the CMC, to equilibration over a large number of time steps (*M*_l_); the new short-time method, which is based on running several shorter duration simulations each started in different initial configurations and evaluating the sample average behavior (*M*_s_).

For the short time scale simulations ten repeats of each sampled system were performed such that we could average the data to minimize noise in the results, except in the case of SLE_3_S where 20 repeats were used to explore noise reduction further. In each case we recorded the nonaggregated surfactant concentration as a function of time along with the number and average size of aggregates to construct a data set.

We used jackknife resampling to measure the variance in this data set. Jackknife resampling involves systematic resampling of the data set to generate subsets by omitting a part of the full data set. The partial set can then can be used to obtain some fit parameters (eq 8 in our case). By aggregating these an estimation of variance in the parameters is possible. Here the jackknife estimator, *j*_ratio_, indicates how much of the original data set is included in each subset.^47,48^ In this study *j*_ratio_ = 0.9 is used throughout, equivalent to a “leave one out” strategy, using 9 out of 10 repeats to construct a subset of data. We also tested *j*_ratio_ = 0.7 and *j*_ratio_ = 0.8 these gave qualitatively similar results but were computationally more demanding since we chose to exhaustively enumerate the subsets. In parallel the average over the complete data set is also obtained.

Both the jackknife subsets and the complete set of data are fitted using the same approach: we define *p*_free_ as the ratio of free surfactant molecules to the total number of surfactant molecules, from our theoretical modeling we expect the long-time kinetics to behave exponentially as in eq 7, so we fit our data accordingly to the exponential

8 $$p_{free}\left(t\right)=A+\left(B-A\right)\exp\left(-\frac{t}{\tau }\right)$$

with fitting parameters *A*, *B*, τ and time *t*. The variation and bounds of these parameters are then obtained by jackknife resampling. To obtain the CMC and the deviation of the CMC the average *A* value and its standard deviation σ_*A*_ are used. Using the method outlined previously by Anderson et al.^8^ it is possible to the calculate the CMC value, since *A* = *p*_free_(∞), and as the total amount of surfactant in the simulation box is known we can obtain *C*_free_ and as such the CMC.

### 4.3 Simulation Details

The dl_meso simulation package was used for all simulations in this study.^49^ As is common in DPD we adopt reduced units, where all DPD beads had unit mass, the temperature *k*_B_ *T* and base length *r*_c_ are set to 1, with the latter representing the cutoff for water bead self-interaction. A DPD time step of 0.01 (reduced DPD time units) was used. Trajectory data snapshots were recorded every 2000 DPD time steps (i.e., 20 DPD time units), and we refer to each snapshot as a “DPD time frame”. A constant pressure (*NPT*) ensemble, was employed using the Langevin piston implementation by Jakobsen.^50^ The pressure was adjusted to match that of pure water in the model, determined separately from pure water bead simulations at *P* = 23.7 ± 0.1 (in DPD units). The Langevin piston relaxation time was set as 100 and the viscosity parameter 0.1. Simulation trajectory file data was analyzed using the ummap analysis package.^51^ Aggregation numbers are extracted as previously described in Anderson et al.^8^ after partitioning the surfactants into submicellar and micellar aggregates as described in earlier sections where the cutoff used to distinguish between the two was set at the aforementioned value n* = 6. All simulations were started from randomly dispersed initial configurations of water beads and surfactant molecules.

Electrostatics associated with the charged DPD beads were introduced through the model of González-Melchor et al.^52^ which assumes a uniform dielectric constant. We chose this to be that of pure water by setting the electrostatic coupling parameter Γ = 15.4. Electrostatics were solved using the smoothed-particle mesh Ewald (SPME) algorithm,^53^ where the number of **k**-vectors are set equal to the simulation box size (to the nearest integer). This ensured the relative error in calculating electrostatic energies was kept below 1%. Each charge was smeared according to a Slater distribution using a smearing length of 1.076 *r*_c_. Such smearing is necessary due to the soft core nature of the beads.

To obtain estimates of the CMC, via the previously established methodology of running long simulations, the following method was used for surfactant–water systems of C_12_E_6_, C_16_E_6_, CAPB, SDS and SLE_3_S at 0.5, 0.75, 1.0 and 1.25% surfactant by weight concentrations. Each simulation was constructed and run for 60,000 DPD time units (90,000 for C_16_E_6_). The simulation times were chosen such that an equilibrium in *C*_free_ was achieved. The concentrations were chosen such that they are well above the experimental CMC but not so high as to form rod-like micelles; only spherical or ellipsoidal micelles were observed. For C_10_E_6_ systems with 0.5, 1.5 and 2.5% surfactant by weight were constructed and run for 60,000 DPD time units. We fixed the number of surfactants in the simulation box at 300 and adjusted the number of water beads to achieve the desired concentration. The decision to use 300 surfactants per simulation is based on a pragmatic choice to ensure that a sufficient number of micelles are able to form while keeping the system size manageable from a computational perspective. In the case of C_16_E_6_ we increase both the number of surfactant molecules in the simulation cell to 900 and the simulation time to 90,000 DPD time units. This was required to achieve good data for this surfactant which has a much lower CMC than the others sampled, too few surfactant molecules lead to depletion of nonaggregated surfactant during the simulation which consequently does not allow for the CMC to be calculated. The CMC is obtained from each individual concentration using eqs 3 or 4 and then averaged across all four to give the final prediction. This longer time scale method will be referred to as *M*_l_.

To test the utility of using multiple short simulations to estimate the CMC, in most cases ten repeat simulations, each starting with a unique random seed, were performed to obtain better data as described in the previous section. Table 1 outlines the simulation details for this further. This shorter time scale method relying on repeat simulations over a shorter time will be referred to as *M*_s_.

**Table 1:** Simulation Details for all Short Time Scale Systems Presented in This Work, Where Molecules Refers to the Number of Surfactant Molecules in a Simulation Box and the Duration is the Simulation Length in DPD Time Units^a^

system	wt %	molecules	duration DPD time units
C_10_E_6_	0.5	300	40k
	0.5	300	40k
	1.5	150	40k
	1.5	300	40k
	1.5	600	40k
	2.5	300	40k
C_12_E_6_	1.00	300	20k
C_16_E_6_	1.00	300	40k
	1.00	600	20k
	1.00	900	20k
CAPB	0.50	300	20k
	0.75	300	20k
	1.00	300	40k
	1.25	300	20k
SDS	1.00	300	20k
SLE_3_S	1.00	300	40k

a In each case 10 repeats are run using different random seeds.

## 5 Results and Discussion

### 5.1 Micelle Growth

In Figure 3 we show (a) the mean aggregation number *n* and (b) the number of micelles *N*_mic_ for each simulated system using method *M*_s_. It is clear that these values have not yet reached an equilibrium value.

**Figure 3:**
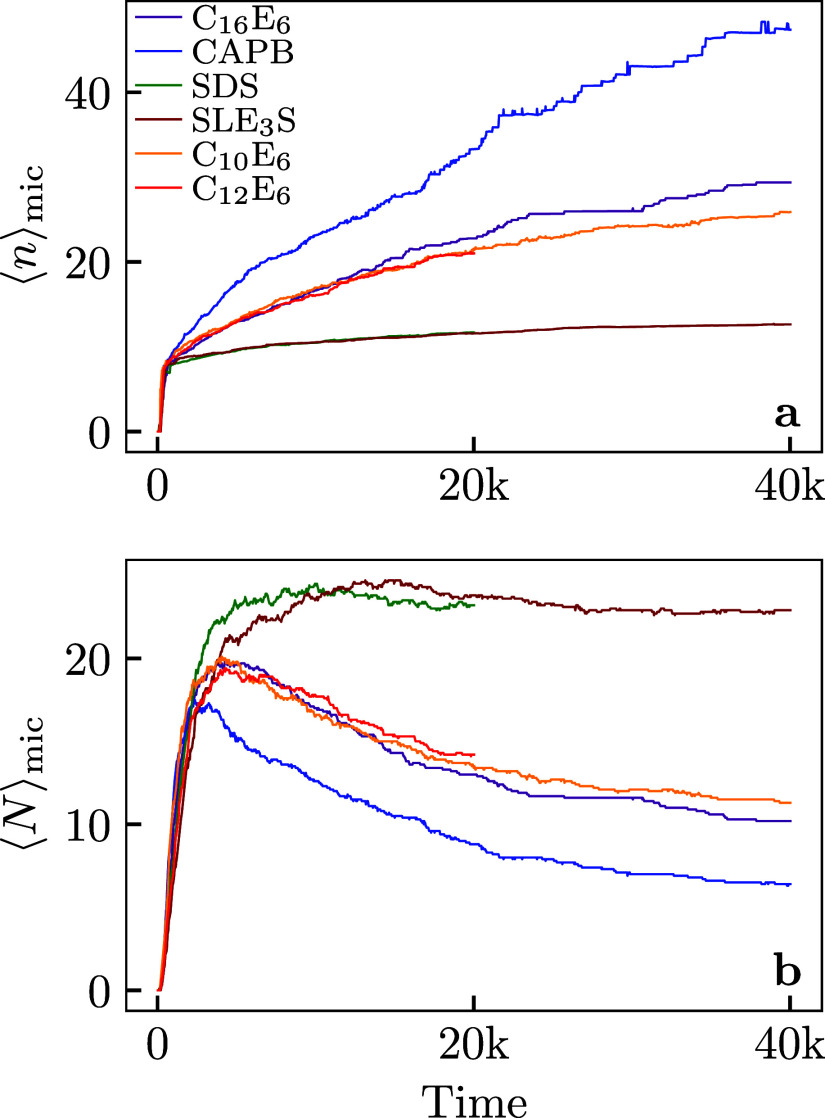
(a) Average aggregation size of micelles ⟨*n*⟩_mic_. (b) Average number of micelles ⟨*N*_mic_⟩ for a set of six different surfactants. Data shown here is the average of 10 random start simulations for each surfactant system. Time is measured in DPD units.

The neutral surfactants (C_16_E_6_, CAPB, C_10_E_6_, C_12_E_6_) show an increasing ⟨*n*⟩_mic_ and a decreasing *N*_mic_ even after *p*_free_ has ceased to vary. By contrast, these quantities vary negligibly for the charged surfactants (SDS and SLE_3_S). These distinct behaviors can be rationalized from our theoretical toy model. The charged surfactants will feature large electrostatic barriers to relaxation which increase with the number of monomers added to an aggregate M . In practice these large barriers would limit the system to single-monomer association/dissociation M=1 . In this limit we found a clear separation between the short-time τ_1_ kinetics and the longer time τ_2_ kinetic which increases *N*_mic_ by decreasing *n*, which is what we see here. The continued evolution of these quantities for the charge neutral systems is presumably because more pathways are possible M>1 . Conversely we can infer that the evolution we observe in the neutral systems corresponds to the initial stages of τ_2_ relaxation. A further piece of evidence for this picture comes from the evolution of the size distribution of micelles shown in Figure 4. Here the log distribution of micelle sizes is tracked as a function of time. For uncharged surfactants the second mode (corresponding to micelles) becomes more sharply defined than for charged surfactants over the same time window. As the slow τ_2_ relaxation enables equilibration in aggregate size, the narrow size distribution of micelle sizes in the charge neutral systems corresponds to a thermal rarefaction. For charged systems the aggregate sizes at the end of τ_1_ are essentially produced by a kinetic process, leading to a broader nonthermal distribution.

**Figure 4:**
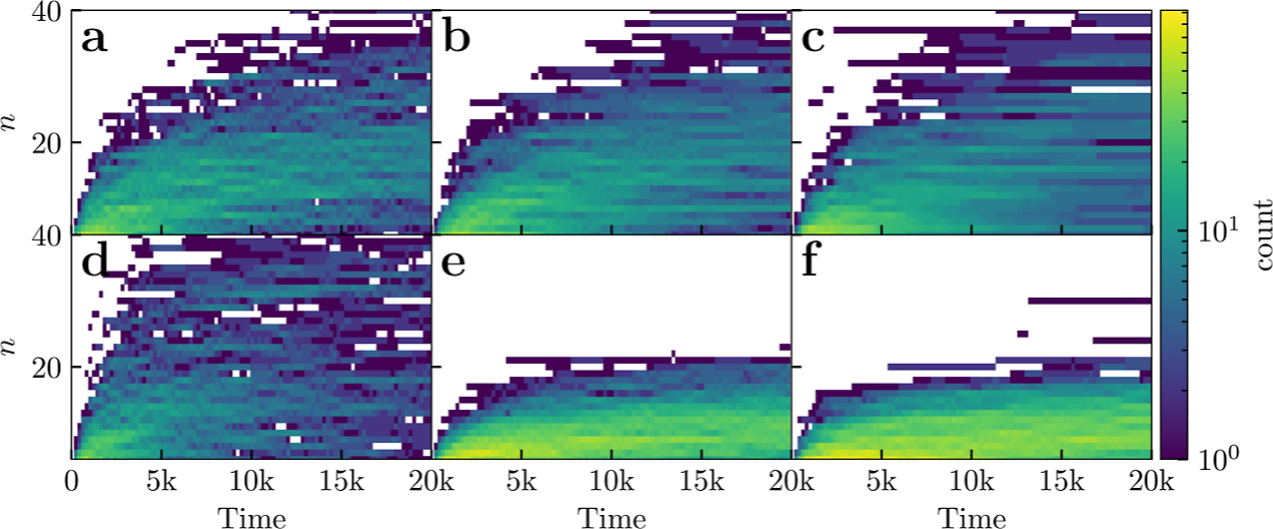
Frequency of the micelle sizes *n*, for (a–d) charge neutral and (e–f) charged surfactants. Specific systems are (a) C_10_E_6_, (b) C_12_E_6_, (c) C_16_E_6_, (d) CAPB, (e) SDS and (f) SLE_3_S. We take the cutoff size for micelles to be n* = 6. All data is averaged across 10 random seed runs. Time is measured in DPD units.

### 5.2 CMC Prediction

In contrast to the behavior of *n* and *N*_mic_, Figure 5a shows the ratio of free surfactant to the total amount of surfactant in the simulation box, *p*_free_, as a function of time for a selection of surfactants. There is decay in *p*_free_(*t*) from the initially pure monomer state *p*_free_(0) = 1 toward a visible finite nonzero plateau value (representing the final equilibrium free monomer population). Suggesting that the monomer concentration settles out before micelle growth (as asserted in Section 3.2). To calculate CMC we only rely on knowing the equilibrated *p*_free_ value and not that of *n* or *N*_mic_. We can obtain the CMC of a system by knowing the concentration of nonaggregated surfactant in the simulation box which we can easily obtain from the *p*_free_ value.

**Figure 5:**
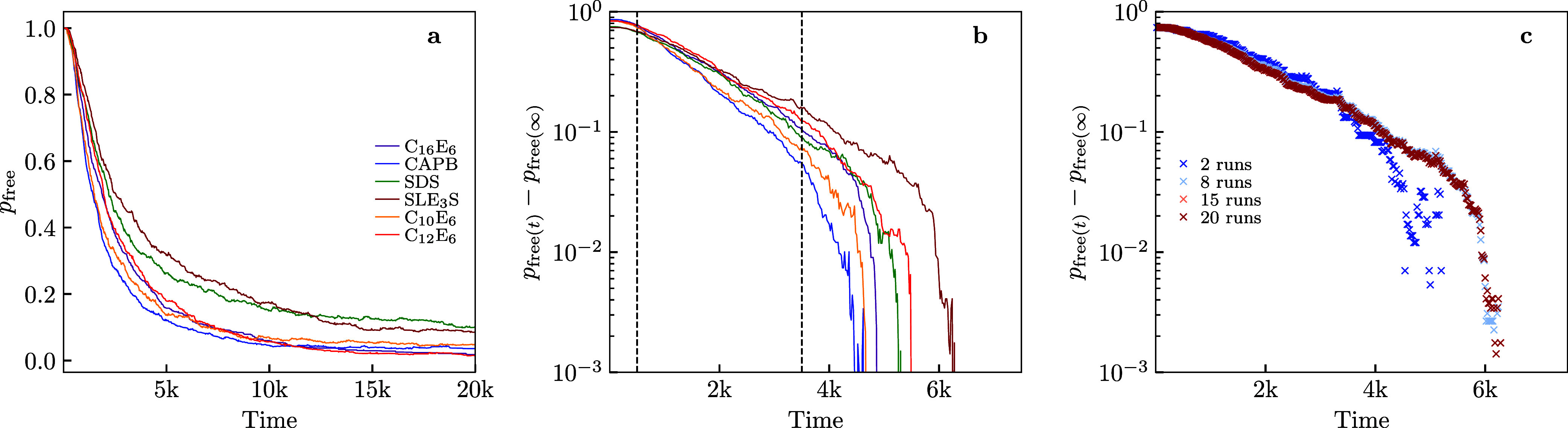
(color online) (a) The ratio of surfactant in a micelle to the total amount of surfactant in the simulation box, *p*_free_. A micelle is any aggregate with at least 6 surfactants. Data is shown for six different types of surfactant, all displaying the same overall trend. Data for each surfactant was obtained by running 10 simulations with different random start points for 20,000 time units and averaging the results. (b) The difference between the free monomer mass ratio, *p*_free_, and the average *p*_free_ taken over the last 20 time units of the simulation for the six different types of surfactant. Dashed vertical lines roughly indicate the linear region. (c) The difference of the free monomer mass ratio, *p*_free_, and the average *p*_free_ taken over the last 20 time units for SLE_3_S, here an increasing number of random seed simulations are used to generate the data (blue to red). Time is given in DPD units.

While running a sufficiently long simulation to equilibrium can be used to estimate the system’s CMC using eqs 3 or ([Disp-formula eq4]), in practice it is challenging to do so as one, it may be difficult to predetermine how long to run out a simulation for an untried surfactant; two, the monomer and micelle exchange rates may be so slow that it takes too long for the micelles to form, grow and merge to an equilibrium state (i.e., very large τ_1_ and τ_2_).

Hence, being able to estimate *p*_free_(∞) from the shorter time scale simulations is advantageous and circumvents the above problems. Our approach taken here is to estimate *p*_free_(∞) via curve fitting *p*_free_(*t*) using only the early times of the simulation and projecting out to long time behavior, this is simply given by *A* in eq eq 8.

Our theoretical model for aggregation predicts an exponential decay toward the plateau value of *p*_free_ in eq 7. We see this behavior in the region between the dashed lines in Figure 5b where the curves of *p*_free_(*t*) – *p*_free_(∞) are straight-lines on log–linear axes. Note that we had to approximate *p*_free_(∞) as the final value of the simulations, so there are deviations from the exponential relationship as this value is approached. The exponential decay becomes increasingly clear with more runs, which is illustrated in Figure 5c. It is not desirable to run hundreds of repeats because this would nullify the computational advantage of the short-time simulation approach, and so we proceed with a pragmatic choice of ten repeats.

We have established that *p*_free_ decays toward a plateau value on the short simulation time scale. The question is whether we can accurately estimate the plateau value and hence CMC using only short simulations. The more detailed dynamics beyond *p*_free_ varies qualitatively depending on whether or not the surfactant is charge neutral (i.e., either nonionic or zwitterionic) in solution.

An exponential decay curve as per eq 8 was fit to the *p*_free_ data for *M*_s_. The curve fit parameter *A* provides an estimate of the plateau value (*p*_free_ at equilibrium, *p*_free_(∞)) and can be used to calculate CMC via eqs 3 or ([Disp-formula eq4]). By systematically truncating the data to short time intervals and obtaining the error in *A* we can explore where the short-time approach is viable for predicting the CMC. To quantify the error in *A* we use method *M*_l_, to measure *p*_free_ at four different concentrations and used the average to compare to the short-time scale approach. We will refer to this as *A*_L_, whereas using the *M*_s_ method, with some fitting window, to obtain the curve fit parameter *A* we will denote with *A*_S_. The mean square error |*A*_L_ – *A*_S_|^2^ obtained in this fashion is shown in Figure 7 and displays a strong dependence on the choice of start and end times for the fitting window. The very high error regions are where the fits yield nonsensical estimates of *A*_S_ < 0 equiv to *p*_free_(∞) < 0. A summary of the results for a varying number of repeats in the short time-scale approach is shown in Table 2 and Figure 6, here we start the fit after 5000 DPD time units and fit for a duration of 10,000 time units in all cases. It is clear that the deviation in the result decreases with more repeats, depending on the desired uncertainty fewer than 10 repeats could be used, though 4 or more are likely desirable.

**Table 2:** Summary of Results for the Indicated Surfactant Systems, Here the Error is Reported for a Varying Numbers of Repeats *m* of Short Simulations Used to Fit *p*_free_, as Well as the *A* Values Obtained Using the Long (*M*_l_) and Short (*M*_s_) Simulation (*m* = 10)

	log_10_(|*A*_L_ – *A*_S_|^2^)					*A* × 10^–2^	
system	*m* = 2	4	6	8	10	*M*_l_	*M*_s_
C_10_E_6_	–3.79	–5.24	–5.44	–5.82	–5.01	5.14	4.82
C_12_E_6_	–3.61	–3.63	–4.19	–1.68	–4.16	0.664	1.50
C_16_E_6_	–4.82	–4.64	–4.64	–4.61	–4.02	0.391	1.37
CAPB	–3.51	–4.08	–3.95	–4.10	–4.10	2.36	3.25
SDS	–1.84	–1.98	–1.97	–2.06	–2.00	9.78	10.4
SLE_3_S	–2.07	–2.08	–2.29	–2.47	–2.46	0.81	6.69

**Figure 6:**
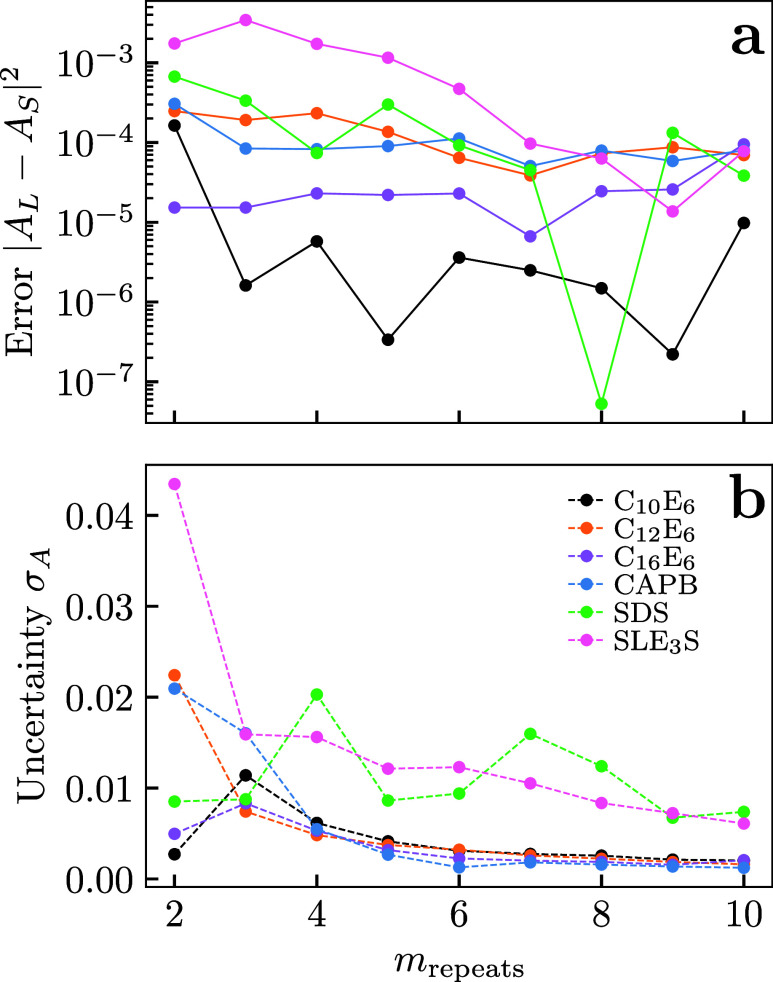
(a) The error between the long and short simulation methodology as a function of *m*_repeats_. (b) The uncertainty in the fit parameter *A*_S_ as a function of *m*_repeats_. Here *m*_repeats_ represents the number of random seed runs used to generate the average data from which *A*_S_ is obtained.

**Figure 7:**
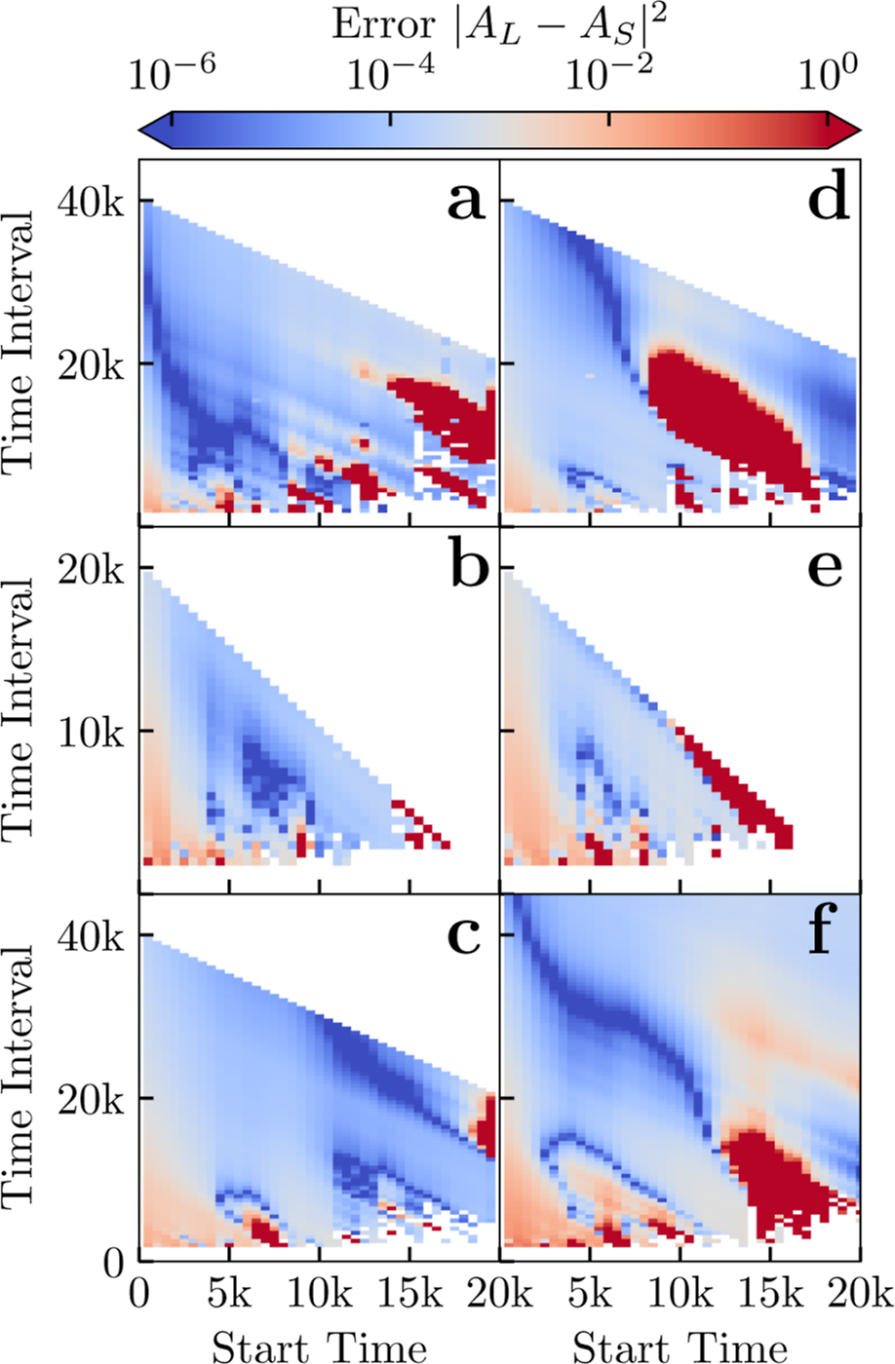
Normalized mean squared error between fitting constant *A*_S_ for all surfactants using 10 repeats compared to the *A*_L_ value predicted using one long simulation at different concentrations above the CMC. (a–d) Charge neutral and (e–f) charged surfactants. Specific systems are (a) C_10_E_6_, (b) C_12_E_6_, (c) C_16_E_6_, (d) CAPB, (e) SDS and (f) SLE_3_S. Time is measured in DPD time units.

The data for the C_*n*_E_*m*_ surfactants (panels (a–c) of Figure 7) shows that the estimate of *A* and as such *p*_free_(∞) and CMC remains of similar level of *absolute* accuracy despite increasing tail length. However, the actual value of *p*_free_ (and the CMC) decreases with tail length, roughly following the well-known Stauff–Klevens rule.^59^ As such, the relative accuracy of |*A*_L_ – *A*_S_|^2^/*A*_S_^2^ actually decreases with increasing tail length. This makes it more difficult and expensive to achieve accurate estimates of *A* with longer surfactants since larger simulation boxes are required for there to be sufficient free surfactant in the simulation box. The data presented here is for a fixed number (300) of surfactants per box. As a rule, we expect larger simulations would be required to achieve similar levels of accuracy for longer tail lengths.

### 5.3 Practical Considerations

To be able to apply this method to new surfactants for which the correct *p*_free_(∞) (i.e., *A*_L_) value is not known, we need to find an alternative to the error surfaces presented in Figure 7 to be able to identify good and bad guesses. Here jackknife resampling is key, as it allows us to obtain an estimate of the goodness of the guess without the long simulation data by determining the deviation in the calculated *A*_*S*_ values. Figure 8 shows that the standard deviation in *A*_S_ obtained through jackknife sampling (right panels) are colocalized with regions of large absolute error (left panels). In principle, jackknife resampling could be used as part of an automated methodology to determine when simulation length is sufficient or when more time is needed for new systems.

**Figure 8:**
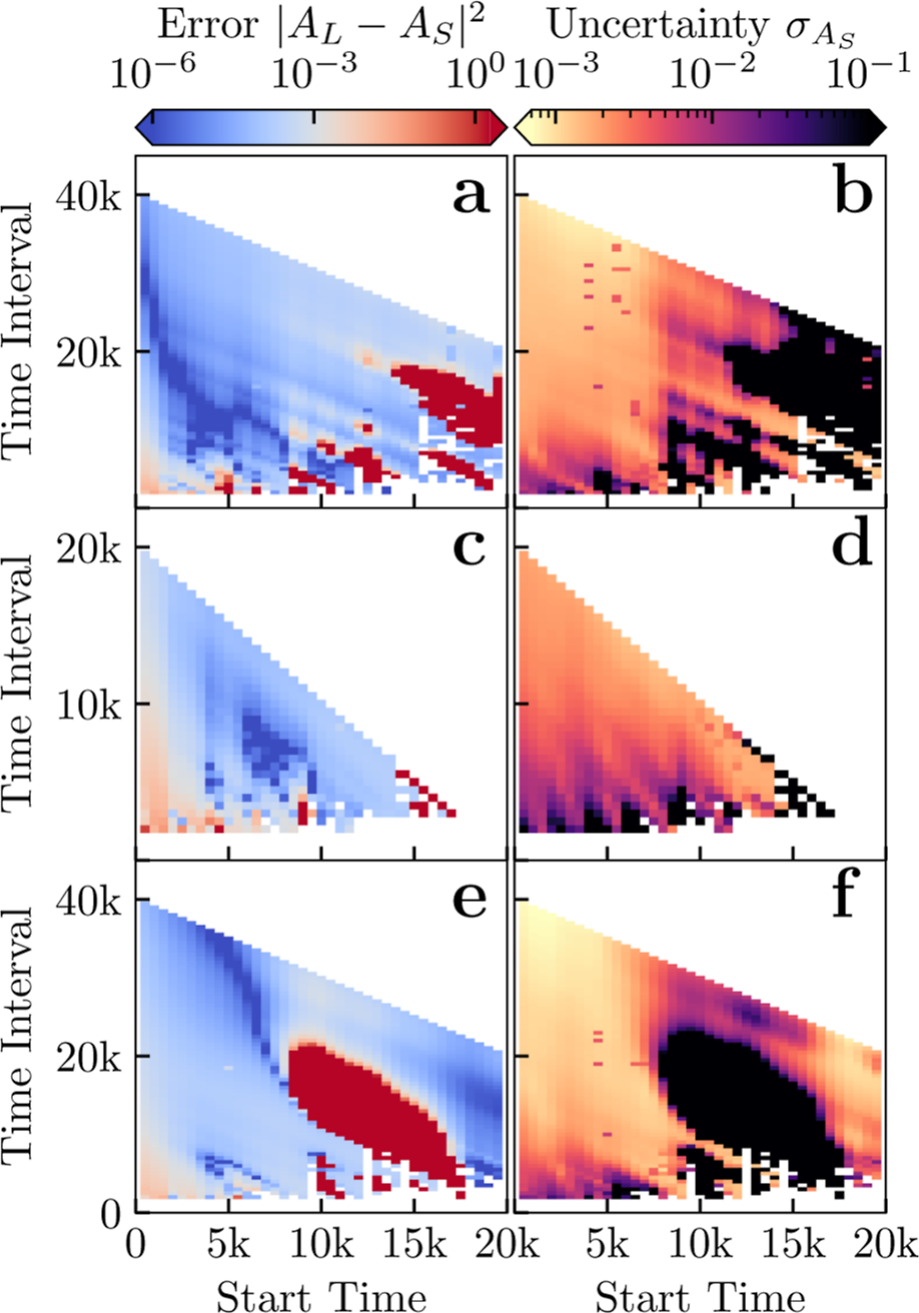
(a,c,e) Mean squared error for fitting constant *A* and (b,d,f) its standard deviation (via jackknife resampling) for (a,b) C_10_E_6_, (c,d) C_12_E_6_ and (e,f) CAPB. The errors in *A*_S_ are compared to the *A*_L_ value predicted using one long simulation at different concentrations above the CMC to obtain an average *A*_L_ value. For jackknife we used 10 repeats. Time is measured in DPD time units.

We note that for each surfactant there is a region of poor fit for short intervals and early start times, presumably due to the transient kinetics of initial micelle formation. The size/shape of this region is system dependent. The time windows where we obtain good estimates of *A* and as such *p*_free_(∞) from our procedure coincides with the time regime over which a smooth distribution around some maxima above the premicellar cutoff emerges. For example comparing CAPB and C_10_E_6_; the latter shows good fits for start times around 2500–7500 time units, using anywhere from 10,000 to 30,000 time units for the fit. From comparing Figure 7 with Figure 4, we can see this corresponds to starting the fit around the time where we see a minima appearing on the micellar size distribution around the premicellar cutoff, the end time shows a clear and smooth distribution around a maxima. CAPB on the other hand has a much less smooth distribution of micelle sizes with no clear maxima, here the distribution while covering a large space of *n* is very “flat”. Our suspicion is that this “flat” distribution emerges from a kinetic process of aggregate-formation operating far-from-equilibrium. On longer time scales, the aggregates are able to begin exploring their free energy landscape giving shape to the distribution as a curved energy funnel. Accordingly, the τ_2_ relaxation becomes thermodynamically constrained. Only over this time scale can we apply our short-time fitting procedure (i.e., an exponential fit) to obtain a good estimate for *p*_free_. For ionic surfactants the distribution of micelle sizes is much more static. We observe here that the initial time required for good fits roughly corresponds to where *p*_free_ has decayed to around 1/*e*. And fitting for a time intervals around 1 to 2 × 10^4^ DPD time units yields good results. The exact time seems to match up to around one decay time in the exponential fit. This conveniently also holds true for the neutral surfactants. These two observations suggests the possibility of automation: simulate the initial kinetics until *p*_free_ has decayed to 1/*e*, then sample until one achieves a self-consistent fit of the time for *p*_free_ to decay to its plateau value with some minimum sampling time (say e.g. 10^4^ time units) to prevent false convergence.

Finally we can investigate the effect of box size and concentration on the predicted *A*_*S*_ value. See Supporting Information for figures. In essence this follows the same observations as above. In order to obtain a good fit we need to have a “funnelled” distribution of micelle sizes (i.e., positive curvature around the modal point). The fit start time should correspond to the time where the number of micelles start to decrease, corresponding to the distribution of micelle sizes shifting to favor larger micelles this roughly corresponds to *p*_free_ having decayed to 1/*e*. The fit end time should still correspond to a smooth distribution of sizes of micelles (usually 10,000–20,000 time units are sufficient). A larger box leads to an overall more “funnelled” distribution of micelle sizes. And a higher concentration leads to a more distinct minima around the premicellar cutoff. Care needs to be taken though as higher concentrations may lead to the formation of rod-like micelles which is not desirable.

The short time scale approach at first glance may appear not much less intensive compared to the previous long simulation approach in terms of computational effort. For example running 10 repeat runs with 20,000 time units is similar to 4 different concentrations run for 60,000 time units. However, there are some additional benefits to having multiple repeat runs, namely should the simulation crash or fail for some reason (which is more likely the longer the simulation is) using say 9 repeats rather than 10 will likely be inconsequential whereas using only 4 different concentrations means each simulation must be run to its conclusion. This may prove particularly expensive for slowly equilibrating systems. Further a surfactant with a low critical micelle concentrations such as C_16_E_6_ often runs into issues when using where very large boxes are needed as otherwise only aggregated surfactant molecules are in the box, this does not allow for the calculation of the CMC, and as such larger more expensive simulations are required. The *M*_s_ method of course also need to consider this but since the simulations here are not run to equilibrium they should remain computationally more efficient, and as several repeat runs are used to obtain averaged data, there should be less numerical fluctuation within the system.

As we have experienced with C_16_E_6_, systems with small CMCs become increasing inaccessible by direct simulation methods, even using the short-time repeat method described here. This is because larger and larger simulation boxes are required to get good statistics, and simultaneously the relaxation times lengthen as the population of free surfactant monomers decreases. In these circumstances, the Stauff–Klevens rule^59^ may come to our rescue, since it suggests the CMC should decrease by an approximately constant factor for each carbon added to the alkyl tail. For example, from Table 3, we infer that the ratio of the CMCs for C_12_E_6_ and C_10_E_6_ is 0.11/1.19 ≃ 0.092. This is therefore an estimate (prediction) of the factor by which the CMC decreases when an extra bead is added to the akyl tail. Extrapolating to C_16_E_6_ (adding three beads) would therefore imply a CMC of 1.19 × (0.092)^3^ ≃ 0.9 × 10^–3^ mM, which is (perhaps serendipitously) close to the experimental value. Thus, in principle we could use this approach to extrapolate the CMC measured for surfactants with shorter alkyl chains to longer alkyl chains, where the latter are inaccessible by direct simulation methods; and indeed it may even be cheaper to simulate a homologous series of short alkyl chain surfactants and extrapolate, rather than attempt a direct simulation of a surfactant with a long alkyl chain. We have not particularly explored this approach beyond noting its obvious feasibility, and it is also something that could in principle be automated.

**Table 3:** Summary of Results for CMC Values in mM for the Various Surfactant Systems, Using the Long and Short Simulation Approaches *M*_l_ and *M*_s_ ^a^

	methodology			
system	*M*_l_	*M*_s_	prev	expt
C_10_E_6_	1.19 ± 0.22	1.71 ± 0.07	0.7	0.90
C_12_E_6_	0.11 ± 0.05	0.32 ± 0.03	0.09	0.082
C_16_E_6_	0.11 ± 0.06	0.25 ± 0.04		0.001
CAPB	1.58 ± 0.77	2.56 ± 0.36		2.9
SDS	4.36 ± 1.64	6.07 ± 1.18	*6.7(1)*	8.2
SLE_3_S	2.38 ± 1.09	3.34 ± 0.77	*2.5(1)*	2.5

a Previous simulation results (penultimate column, italics) are from ref 7 for C_10_E_6_ and C_12_E_6_, from ref 8 for SDS, and from ref 9 for SLE_3_S. Experimental values (final column) are taken from refs 54,55 for C_10_E_6_ and C_12_E_6_, from ref 54 for C_16_E_6_, from ref 56 for CAPB, and from refs 57,58 for SDS and SLE_*m*_S.

In our previous DPD simulation work using varying concentrations of ionic surfactants, in addition to surfactant mixtures,^9^ we also observed exponential decay for all systems. As such the method presented here should be suitable for surfactant blends. The sampled concentrations simply need to be chosen to be above the CMC at a concentration where micelles are present and spherical in shape, i.e., the concentration should not be so high that rod-like micelles (or other mesophases) are formed.

## 6 Conclusions

We have demonstrated the viability of short-time simulations for estimating the CMC of model surfactant systems, albeit with several important caveats. We have done this by fitting *p*_free_, the ratio of nonaggregated to aggregated surfactant, to a theory derived exponential decay equation to predict the final *p*_free_(∞) value from the fitting constant *A*. We can directly obtain the concentration of free surfactant, *C*_free_, from this and as such the CMC.

The short-time simulations can yield good estimates of the CMC as shown in Table 2, but this procedure is very sensitive to the time window in which is sampled as shown in Figure 7. Starting the fit around 5000 time units and fitting for 10,000 time units gives a reasonable result for *p*_free_, with at least 5 repeats giving a low deviation in the result, thus requiring 5 simulation of 15,000 time units, which is significantly cheaper than the long run method, requiring typically around 4 simulations of 60,000 time units. For charged surfactants slightly longer fit times are required.

We determined that for an ideal system we want to see a swift move away from its initial monomer-centric distribution with some bell-shaped distribution of micelle size around some maximum well above the premicellar cutoff. A “flat” or uneven distribution does not lend itself for fitting. The choice of concentration should be such that we are well above the CMC without seeing nonspherical micelles. The choice of box size is a compromise of seeing a smooth distribution of micelle sizes and computational cost. The deviation in *A* can be used to indicate bad regions for the fit, as such making it easy to avoid them. Good regions typically appear when the fit is started soon after we see distribution shift away from its initial configuration but before having reached a quasi equilibrium state. A good rule of thumb was found to be to simulate the initial kinetics until *p*_free_ has decayed to 1/*e*, then sample until one achieves a self-consistent fit of the time for *p*_free_ to decay to its plateau value. Generally quicker kinetics require fewer fitted time units. Similarly larger boxes and higher concentrations also favor shorter fits and earlier start times. We also note that it may be cheaper to simulate a homologous series of short alkyl chain surfactants and extrapolate using the Stauff–Klevens rule, rather than attempt a direct simulation of a surfactant with a long alkyl chain.

Finally, we note that our approach heavily relies on an exponential fit of the initial aggregation formation kinetics. Underlying this fit is a rather simplified model for the aggregation kinetics and micelle thermodynamics. In principle this procedure could be refined with more sophisticated theories that extend the method’s viability. However, as it stands the method provides good results for a wide range of systems.

## Supplementary Material



## Notes

DPD parameters used for the systems presented in this work, information on how bonded and nonbonded interactions are handled and finally the effect of box size and surfactant concentration on CMC predictions and micelle size distributions (PDF)
