# Supplementary material for: Predicting Critical Micelle Concentrations from Short Time Scale Simulations
Source: J Phys Chem B. 2025 Dec 17;130(1):618–29. doi: 10.1021/acs.jpcb.5c07475 (PMC12794194; doi:10.1021/acs.jpcb.5c07475)
Supplement: Supplementary file 1 [file jp5c07475_si_001.pdf]

Supporting Information - Predicting Critical Micelle Concentrations from Short Timescale Simulations

Felix Rummel,*,† Joshua F. Robinson,† Patrick B. Warren,† David J. Bray,† and Richard L. Anderson*,†

†*The Hartree Centre, STFC Daresbury Laboratory, Warrington, WA4 4AD, United Kingdom*

‡*H. H. Wills Physics Laboratory, University of Bristol, Bristol BS8 1TL, United Kingdom*

E-mail: felix.rummel@stfc.ac.uk; richard.anderson@stfc.ac.uk

A DPD simulation methodology & model parameters

In this section we discuss the adopted DPD method in more detail than presented in the main article.

Table S1: DPD repulsion amplitudes and cut-off distances

bead i	bead j	A_{ij}	R_{ij}	bead i	bead j	A_{ij}	R_{ij}
CCONC	CCONC	22	1.218	CH_3	CH_2OCH_2	28.5	1.0355
CCONC	CH_2	32	1.0715	CH_3	$CH_2OSO_3{}^-$	28.5	1.0945
CCONC	COO^-	18.6	1.104	CH_3	Na^+	45	0.9775
CCONC	$CNMe_2C^+$	11	1.3165	CH_3CH_2	CH_3CH_2	22	1.096
CH_2	CH_2	24	0.925	CH_3CH_2	CH_2OH	26	1.038
CH_2	COO^-	28.5	0.9575	CH_3CH_2	CH_2OCH_2	28.5	1.106
CH_2	$CNMe_2C^+$	15.6	1.17	CH_3CH_2	CCONC	32	1.157
CH_2CH_2	CH_2CH_2	22	1.074	CH_3CH_2	CH_2	23	1.0105
CH_2CH_2	CH_3CH_2	24	1.085	CH_3CH_2	COO^-	39.5	1.043
CH_2CH_2	CH_2OH	26	1.027	CH_3CH_2	$CNMe_2C^+$	22.7	1.2555
CH_2CH_2	CH_2OCH_2	28.5	1.095	$CNMe_2C^+$	$CNMe_2C^+$	8.8	1.415
CH_2CH_2	$CH_2OSO_3{}^-$	28.5	1.154	COO^-	COO^-	25.8	0.99
CH_2CH_2	Na^+	45	1.037	COO^-	$CNMe_2C^+$	14.4	1.2025
CH_2CH_2	CCONC	32	1.146	$2\,H_2O$	$2\,H_2O$	25	1
CH_2CH_2	CH_2	23	0.9995	$2\,H_2O$	CH_3	45	0.9775
CH_2CH_2	COO^-	40.9	1.032	$2\,H_2O$	CH_2CH_2	45	1.037
CH_2CH_2	$CNMe_2C^+$	23.3	1.2445	$2\,H_2O$	CH_3CH_2	45	1.048
CH_2OCH_2	CH_2OCH_2	25.5	1.116	$2\,H_2O$	CH_2OH	14.5	0.99
CH_2OCH_2	$CH_2OSO_3{}^-$	15.4	1.175	$2\,H_2O$	CH_2OCH_2	24	1.058
CH_2OCH_2	Na^+	24	1.058	$2\,H_2O$	$CH_2OSO_3{}^-$	17.9	1.117
CH_2OH	CH_2OH	14	0.95	$2\,H_2O$	Na^+	25	1
CH_2OH	CH_2OCH_2	25	1.048	$2\,H_2O$	CCONC	19	1.109
$CH_2OSO_3{}^-$	$CH_2OSO_3{}^-$	13.3	1.234	$2\,H_2O$	CH_2	45	0.9625
$CH_2OSO_3{}^-$	Na^+	17.9	1.117	$2\,H_2O$	COO^-	25.4	0.995
CH_3	CH_3	24	0.955	$2\,H_2O$	$CNMe_2C^+$	14.2	1.2075
CH_3	CH_2CH_2	23	1.0145	Na^+	Na^+	25	1
CH_3	CH_2OH	26	0.9675				

A.1 Non-bonded interactions

The non-bonded interactions are defined by standard DPD pairwise soft repulsions,[1,2] of the form $U_{ij} = \frac{1}{2}A_{ij}R_{ij}(1 - r_{ij}/R_{ij})^2$ for $r_{ij} \leq R_{ij}$ and $U_{ij} = 0$ for $r_{ij} > R_{ij}$, where A_{ij} is the repulsion amplitude of the DPD conservative force, R_{ij} the cut-off distance, and $r_{ij} = |\vec{r}_j - \vec{r}_i|$ the separation between beads i and j located at $\vec{r}_i$ and $\vec{r}_j$ respectively. Solvent beads have a $A_{ij} = 25$ and $R_{ij} = 1$, where each DPD distance unit is 5.64 Å.[3]

As in our previous works,[3–6] we use the self-interaction cut-offs R_{ii} to capture the contribution of the molecular fragments to the overall molar volumes (here and below i denotes bead type, not individual beads). For this, the Durchschlag and Zipper rules were used to assign R_{ii}^3 values for different beads in proportion to their fragment molar volumes,[7] taking the molar volume of water as a reference. Then, between dissimilar bead types we use a R_{ij} for dissimilar bead types are obtained via simple arithmetic 'mixing rule' of $R_{ij} = (R_{ii} + R_{jj})/2$.

For the repulsion amplitudes we adopt the values of A_{ij} determined in our earlier work, obtained by fitting to experimental water-octanol partition coefficients ($\log P$ values), mutual solubilities, and liquid density data, for a range of small molecules. These parameters have previously been applied to the exploration of micelle properties, phase behaviour and scission energies associated with a variety of surfactants.[3–6,8,9]

The charged DPD beads in the present work are assigned unit charges ± 1 according to their formal physical charge. In DPD though, charge smearing is usually included to remove singularities in the electrostatic interactions.

Here we use Slater-type charge smearing proposed by González-Melchor *et al.* in which the Coulomb potential for pairs of point charges is modified to eliminate the Coloumb divergence at contact.[10] For the electrostatic pair potential therefore,

$$U_{ij}^E(r_{ij}) = \frac{\Gamma q_i q_j}{4\pi r_{ij}}[1 - (1 + \beta r_{ij})e^{-2\beta r_{ij}}], \tag{1}$$

where r_{ij} is the ion separation, q_i and q_j are the ion charges (valencies), $\Gamma = e^2/(k_B T \epsilon_0 \epsilon_r r_c)$

is a dimensionless electrostatic coupling parameter which includes the relative background permittivity, and β is a tuneable Slater smearing parameter. For aqueous systems with $r_c \approx 5.6\,\text{Å}$ as studied here, $\Gamma \approx 15$.

Since the modified Coulomb interaction retains the long-range $1/r$ dependence, it is essential to account for the periodic boundary conditions. An appropriate approach is the Ewald summation method,[11] in which the total electrostatic energy is

$$
\begin{aligned}
U^E_{total} = \frac{\Gamma}{4\pi}\Big[\sum_i \sum_{j>i} \frac{q_i q_j}{r_{ij}}[\operatorname{erfc}(\alpha r_{ij}) - (1+\beta r_{ij})e^{-2\beta r_{ij}}] \\
+ \frac{2\pi}{V}\sum_{\vec{k}\neq 0} \frac{e^{-k^2/4\alpha^2}}{k^2} S(\vec{k})S(-\vec{k}) - \frac{\alpha}{\sqrt{\pi}}\sum_i^N q_i^2\Big]\,.
\end{aligned} \tag{2}
$$

In this α is the parameter controlling the labor division between real and reciprocal space, $\vec{k}$ are reciprocal space vectors concomitant with the periodic boundary conditions, and $S(\vec{k}) = \sum_i q_i e^{i\vec{k}\cdot\vec{r}_i}$ is the charge structure factor. The only change to the 'vanilla' Ewald method required due to charge smearing lies in the real space contribution. In this case, the associated pairwise short-range electrostatic force between beads i and j is

$$
\begin{aligned}
\vec{F}^{E,S}_{ij} = \frac{\Gamma q_i q_j}{4\pi r_{ij}^2}\Big[\operatorname{erfc}(\alpha r_{ij}) + \frac{2\alpha r_{ij}}{\sqrt{\pi}} e^{-\alpha^2 r_{ij}^2} \\
- e^{-2\beta r_{ij}}[1 + 2\beta r_{ij}(1+\beta r_{ij})]\Big]\,.
\end{aligned} \tag{3}
$$

Any standard method for dealing with the reciprocal space part of the Ewald sum, such as Smooth Particle Mesh Ewald (SPME),[12] can be used without modification (including the associated expressions for reciprocal space force $\vec{F}^{E,L}_i$). For optimisation, a study of truncation effects due to choice of the values of α, β and the real-space cutoff is given by Vaiwala *et al.*[13] In the case of SPME, the charge interpolation order is an additional parameter: this can be carefully selected to ensure the reciprocal space Ewald interactions are applied sufficiently accurately.

A full list of the DPD conservative interaction parameters, A_{ij}, is presented in Table S1.

The cut-off distance for the dissipative and random forces required in the DPD method was assigned equal to the maximum cut-off distance in the system (*i. e.* set as the maximum value of R_{ii}) and the dissipative friction amplitude was set at $\gamma = 4.5$.

A.2 Bonded interactions

A simple harmonic potential $U_{ij}^B = \frac{1}{2}k_b(r_{ij} - r_0)^2$ is used for the bonds between connected DPD beads. As in our earlier work,[4] nominal bond lengths are set according to the combined number of heavy atoms found along the main chain of the beads and a single bond constant $k_b = 150$ (DPD units) was adopted throughout. For CH_2CH_2 bonded pairs this results in $r_0 = 0.39$. In real units this corresponds to 2.2 Å. Note that in our approach the standard DPD (non-bonded) replusions are retained between all beads, including directly bonded pairs. This has the effect of increasing the bond length (in addition to fluctuation and correlation effects) above the nominal value indicated by r_0. Thus between pairs of bonded CH_2CH_2 beads this results in a mean bond length of 0.49 DPD units, or 2.7 Å. Corrections to the r_0 values can be determined using Eq. 1 of the supporting information from Bray *et al.*[14], or from direct measurement from simulation. For CH_2CH_2 pairs, this value is in good alignment with known bond lengths. In the present work we do not refine the r_0 values as to do so would significantly impact the underlying parametrization based upon fitting to liquid densities and $\log P$ values.[4]

We introduce bond rigidity by including a harmonic angular potential between pairs of bonds. We adopt the three-body angular potential used by Venturoli *et al.*,[15,16] *viz.* $U_{ijk}^A = \frac{1}{2}k_a(\theta_{ijk} - \theta_0)^2$ where θ_{ijk} is the angle between adjoining bonds. Here we used $\theta_0 = 180°$ and $k_a = 5$ (DPD units) for all angles.

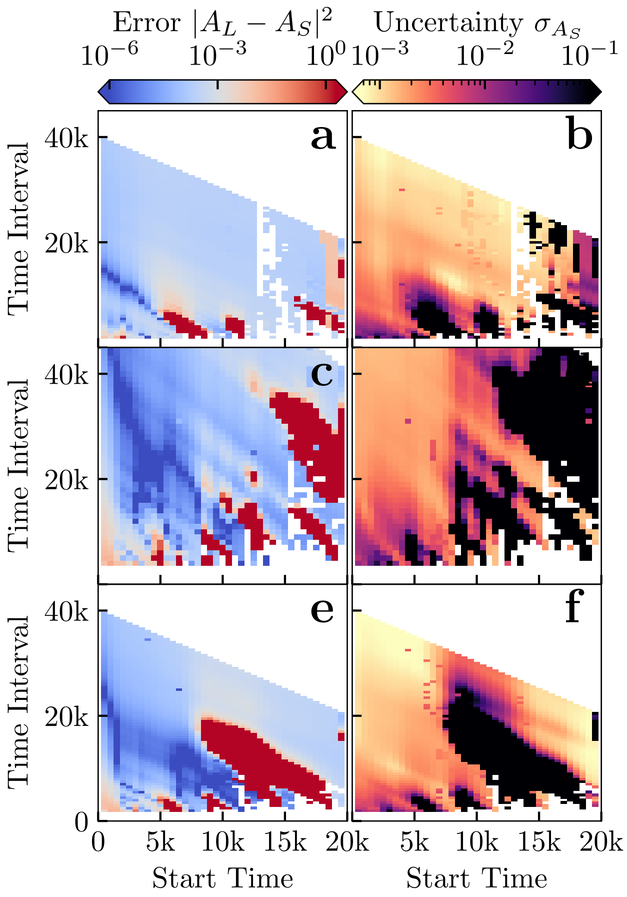

Figure S1: (a,c,e) Mean squared error for fitting constant A and (b,d,f) its standard deviation (via jackknife resampling) for $C_{10}E_6$ with (a-b) 150 surfactants $L^3 = 34^3$, (c-d) 300 surfactants $L^3 = 43^3$ and (e-f) 600 surfactants $L^3 = 54^3$. Where L^3 specifies the dimensions of the cubic simulation box in DPD units. The errors in A are compared to the p_{free} value predicted using one long simulation at different concentrations above the CMC to obtain an average p_{free} value. For jackknife we used 10 repeats. Time is measured in DPD time units.

B Concentration and box-size effects

Figure S1 shows the effect of box size and Fig. S2 the effect of concentration on the prediction with Fig. S3 showing the distribution of aggregation sizes for these simulations. For concentration effects the size distribution is 'flattest' for 1.5 wt %, with it still having a clear maximum. This concentration also has the quickest move away from it's initial distribution further suggesting it to be better for the fitting strategy. Similarly for box size it is clear that a smoother distribution as seen in the larger boxes yields consistently better fitting results.

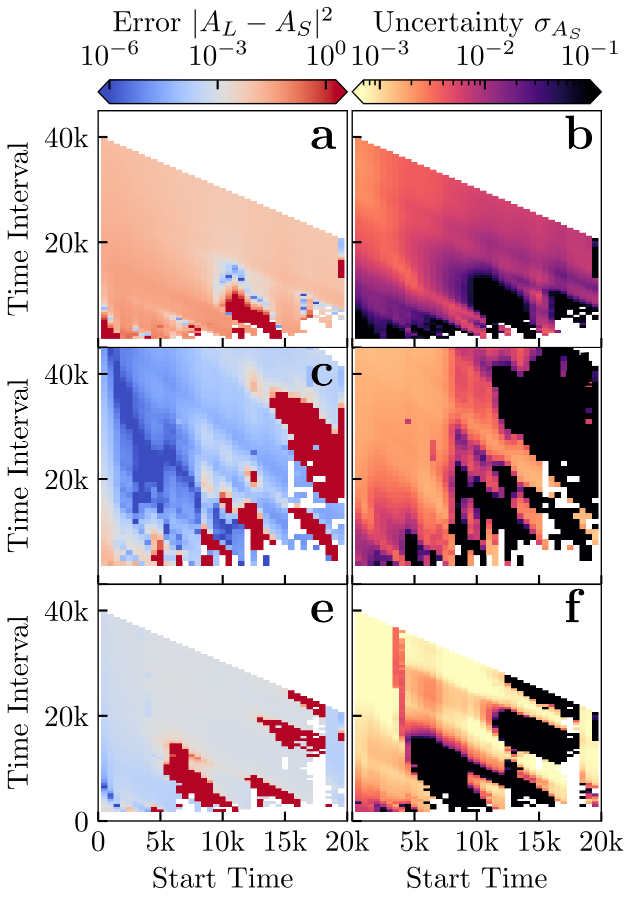

Figure S2: (a,c,e) Mean squared error for fitting constant A and (b,d,f) its standard deviation (via jackknife resampling) for $C_{10}E_6$ with (a-b) 0.5 wt %, (c-d) 1.5 wt % and (e-f) 2.5 wt %. The errors in A are compared to the p_{free} value predicted using one long simulation at different concentrations above the CMC to obtain an average p_{free} value. For jackknife we used 10 repeats. Time is measured in DPD time units.

Of course this is computationally more expensive. Using 300 rather than 600 surfactants per box also appears to have a faster shift away from its initial distribution and no sudden jumps, whereas in the larger box it appears we see some micelle-micelle combination since there is a sudden appearance in micelles with a size of circa 50.

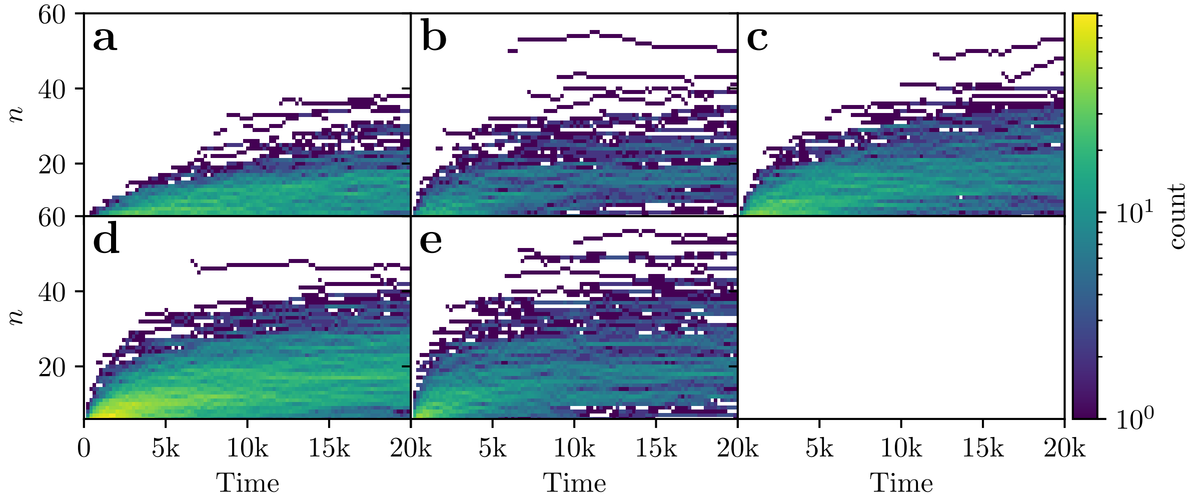

Figure S3: Log frequency plot of the micelle size distribution for $C_{10}E_6$, with various concentrations and number of surfactant molecules per simulation box. All data is averaged across 10 random seed runs. The concentration and number of surfactants in a box are: (a) 0.5 wt % & 300, (b) 1.5 wt % & 150, (c) 1.5 wt % & 300, (d) 1.5 wt % & 600, (e) 2.5 wt % & 300. Time is measured in DPD units

References

(1) Groot, R. D.; Warren, P. B. Dissipative particle dynamics: Bridging the gap between atomistic and mesoscopic simulation. *J. Chem. Phys.* **1997**, *107*, 4423–4435.

(2) Español, P.; Warren, P. B. Perspective: Dissipative particle dynamics. *J. Chem. Phys.* **2017**, *146*, 150901.

(3) Anderson, R. L.; Bray, D. J.; Del Regno, A.; Seaton, M. A.; Ferrante, A. S.; Warren, P. B. Micelle formation in alkyl sulfate surfactants using dissipative particle dynamics. *J. Chem. Theory Comput.* **2018**, *14*, 2633–2643.

(4) Anderson, R. L.; Bray, D. J.; Ferrante, A. S.; Noro, M. G.; Stott, I. P.; Warren, P. B. Dissipative particle dynamics: systematic parametrization using water-octanol partition coefficients. *J. Chem. Phys.* **2017**, *147*, 094503.

(5) Del Regno, A.; Warren, P. B.; Bray, D. J.; Anderson, R. L. Critical Micelle Concentrations in Surfactant Mixtures and Blends by Simulation. *J. Phys. Chem. B* **2021**, *125*, 5983–5990.

(6) Panoukidou, M.; Wand, C. R.; Del Regno, A.; Anderson, R. L.; Carbone, P. Constructing the phase diagram of sodium laurylethoxysulfate using dissipative particle dynamics. *J. Coll. Interf. Sci.* **2019**, *557*, 34–44.

(7) Durchschlag, H.; Zipper, P. Calculation of the partial volume of organic compounds and polymers. *Prog. Coll. Polym. Sci.* **1994**, *94*, 20–39.

(8) Anderson, R. L.; Gunn, D. S. D.; Taddese, T.; Lavagnini, E.; Warren, P. B.; Bray, D. J. Phase Behavior of Alkyl Ethoxylate Surfactants in a Dissipative Particle Dynamics Model. *J. Phys. Chem. B* **2023**, *127*, 1674–1687.

(9) Wand, C. R.; Panoukidou, M.; Del Regno, A.; Anderson, R. L.; Carbone, P. The Relationship between Wormlike Micelle Scission Free Energy and Micellar Composition:

The Case of Sodium Lauryl Ether Sulfate and Cocamidopropyl Betaine. *Langmuir* **2020**, *36*, 12288–12298.

(10) González-Melchor, M.; Mayoral, E.; Velázquez, M. E.; Alejandre, J. Electrostatic interactions in dissipative particle dynamics using the Ewald sums. *J. Chem. Phys.* **2006**, *125*, 224107.

(11) Frenkel, D.; Smit, B. *Understanding Molecular Simulation*; Academic Press: San Diego, CA, 2001.

(12) Essmann, U.; Perera, L.; Berkowitz, M. L.; Darden, T.; Lee, H.; Pedersen, L. G. A smooth particle mesh Ewald method. *J. Chem. Phys.* **1995**, *103*, 8577–8593.

(13) Vaiwala, R.; Jadhav, S.; Thaokar, R. Electrostatic interactions in dissipative particle dynamics—Ewald-like formalism, error analysis, and pressure computation. *J. Chem. Phys.* **2017**, *146*, 124904.

(14) Bray, D. J.; Anderson, R. L.; Warren, P. B.; Lewtas, K. Wax formation in linear and branched alkanes with dissipative particle dynamics. *J. Chem. Theory Comput.* **2020**, *16*, 7109–7122.

(15) Venturoli, M.; Smit, B. Simulating the self-assembly of model membranes. *Phys. Chem. Comm.* **1999**, *2*, 45–49.

(16) Venturoli, M.; Sperotto, M. M.; Kranenburg, M.; Smit, B. Mesoscopic models of biological membranes. *Phys. Rep.* **2006**, *437*, 1–54.
